# Paroxysmal Hypnogenic Dyskinesia Responsive to Doxylamine: A Case Report

**DOI:** 10.1155/2012/484689

**Published:** 2012-07-16

**Authors:** Daniel M. Williams

**Affiliations:** Department of Psychiatry, Scott & White Healthcare, 2401 South 31st Street, Temple, TX 76508, USA

## Abstract

Paroxysmal hypnogenic dyskinesia is a rare clinical entity characterized by intermittent dystonia and choreoathetoid movements that begin exclusively during sleep, often with consciousness preserved once the patient is awakened during the episodes. They occur almost every night and are often misdiagnosed as sleeping disorders. Paroxysmal hypnogenic dyskinesia is currently known to be a form of frontal lobe epilepsy, but not in all cases. We present a 19-year-old male patient with paroxysmal hypnogenic dyskinesia who responded to antihistamines. This supports an alternative theory from 1977 (before the cases had been adequately described) that the disorder lies in dysregulation in the basal ganglia. This description now appears similar to acute dystonic reactions such as extrapyramidal symptoms from antipsychotic medications, which also respond to antihistamines.

## 1. Introduction 

Paroxysmal hypnogenic dyskinesia is a rare clinical entity characterized by intermittent dystonia and choreoathetoid movements that begin exclusively during sleep, often with consciousness preserved once the patient is awakened during the episodes. They occur almost every night and are often misdiagnosed as sleeping disorders. Paroxysmal hypnogenic dyskinesia is currently known to be a form of frontal lobe epilepsy, but not in all cases.

## 2. Case Presentation

A 19-year-old healthy, Caucasian man presented with a two-year history of nonrestful sleep nearly every night. He was referred by his employer for evaluation. Nine months prior to presentation, he learned for the first time from coworkers that he shook frequently while sleeping. He had one episode of being awoken to find his extremities moving uncontrollably for a few minutes. These events continued nearly every night, presumably as they had before. There were never any nightmares or terrors, other than anxiety from not having control of his body several times upon awakening. He had a normal polysomnography test at that time.

This condition prevented him from deploying with his military unit, which greatly saddened him. He discovered that Doxylamine (Unisom), an H1 receptor antagonist, alleviated his symptoms, provided restful sleep, and decreased muscle fatigue in the mornings 100% of the time. Hypothermia, sleep deprivation, extreme exercise, explosions, and stress had never triggered an event. He had no family history of this condition and a completely normal physical exam.

The patient was admitted to the Epilepsy Monitoring Unit at Texas A&M Health Science Center at Scott & White Hospital for 96 hours of continuous EEG-video monitoring. The patient had a total of four dyskinetic events during slow-wave sleep, two of which were precipitated by external stimuli (knocking on the door and turning on the light with voice). The episodes lasted approximately 40 seconds total and each began with 3–6 seconds of a tonic phase of both upper extremities and truncal flexors, sometimes partially sitting upright. Choreoathetoid movements followed, which involved primarily the upper extremities, most notably in the wrists and fingers. It appeared as if the patient were trying to get something off or way from his person, primarily towards the left. He also had intermittent torticollis to the right. The episodes ended with the patient laying down appearing to try and get comfortable in the bed, fidgeting with the blanket, quickly moving from his left and right sides, with facial grimacing and choreoathetoid movements of his head, neck, and hands as the movements minimized over approximately 15 seconds. Sleep appeared to resume quite suddenly.

During one of the episodes, staff carried on a conversation with him in which he was oriented to himself, place, and time. He was aware of what was happening to his body and was able to confirm that the ongoing episode was typical for the episodes he has been aware of in the past. He was not confused at all when the episode ended and remained awake talking with the staff.

## 3. Discussion

There are four major classifications of paroxysmal choreoathetoid dyskinesias: (1) paroxysmal hypnogenic dyskinesia (PHD) (occurs during sleep); (2) paroxysmal kinesigenic dyskinesia (induced by sudden movement); (3) paroxysmal nonkinesigenic dyskinesia (occurs spontaneously when awake); (4) paroxysmal exertion-induced dyskinesia.

So far, our emerging knowledge of the underlying genetic predisposition for these disorders does not account for the spontaneous, nonfamilial cases that have been described elsewhere [1]. The prevailing theory is that paroxysmal hypnogenic dyskinesia results from mesial frontal lobe epilepsy with an autosomal dominant inheritance pattern (ANFLE gene), albeit a heterogenous genetic condition [2]. Less than 100 cases of PHD have been described, making PHD the rarest category of the paroxysmal choreothetoid dyskinesa [3]. In the largest epidemiologic study of this disorder, a questionnaire mailed to 229 medical institutions in Japan revealed only 150 patients with paroxysmal choreoathetoid dyskinesias; only one had PHD [4]. What is known is that there are considerable genetic variations and perhaps other causes that can lead to the same clinical syndrome [1]. For example, mutations of neuronal nicotinic acetylcholine receptor gene (NaChR) on chromosome 20 q have been reported in some families with ANDFLE, and mutations also occur on chromosome 15 [1, 5]. Voltage-gated ion channels have been implicated as a physiologic cause for paroxysmal nonkinesigenic dyskinesia as well [6].

However, while this understanding helped us to clarify it as a distinct clinical entity, the debate regarding the possibility of different proximate causes leading to the same syndrome has continued since the 1970s [7–9]. Bhatia [10] clearly outlined the arguments for a cortical versus a subcortical focus as the cause of PHD: “The arguments in favor of a subcortical focus and not a cortical one are the absence of seizure discharges on EEG in the majority of cases, the absence of evolution of the attacks into generalized or focal convulsions and the lack of an associated loss of consciousness or amnesia.”

Arguments in favor of a basal ganglia disease are the clinical characteristics of the involuntary movements, the absence of EEG abnormalities during the attacks, the occurrence of symptomatic PKC in conditions known to affect basal ganglia function, and the lack of family or past history of epilepsy [10–12]. 

Various pharmacotherapies for the four categories of paroxysmal choreoathetoid dyskinesias have been published, but PHD has received little attention because it is the rarest of the rare. Carbamazepine and other anticonvulsants appear to be very effective in most cases of paroxysmal kinesigenic dyskinesia [13, 14]. Paroxysmal exercise-induced dystonia does not appear to respond reliably to anticonvulsants, levodopa, acetazolamide, or trihexyphenidyl [10].

Here, we present a patient with PHD who responded to antihistamines, supporting an alternative theory from 1977 (before the cases had been adequately described) that the disorder lies in dysregulation in the basal ganglia [15]. Lance [8] suggested that occasional dystonic and choreoathetotic symptoms may be due to a “hereditary sensitivity to dopamine or some related transmitter and that stress, excitement and any situation in which norepinephrine is liberated allows the build-up of its precursor, dopamine, in the basal ganglia in a concentration sufficient to cause paroxysmal dystonia.” This description now appears similar to acute dystonic reactions such as extrapyramidal symptoms from antipsychotic medications, which also respond to antihistamines [8]. 

Considering the rarity of PHD, statistically significant data supporting treatment algorithms may never develop. A trial of antihistamines seems appropriate in patients with PHD or any of the paroxysmal choreoathetoid dyskinesias who fail to respond to anticonvulsants.
